# Construction of the descriptive system for the assessment of quality of life AQoL-6D utility instrument

**DOI:** 10.1186/1477-7525-10-38

**Published:** 2012-04-17

**Authors:** Jeffrey RJ Richardson, Stuart J Peacock, Graeme Hawthorne, Angelo Iezzi, Gerald Elsworth, Neil A Day

**Affiliations:** 1 Centre for Health Economics, Monash University, Wellington Road, Clayton, VIC 3800, Australia; 2 Canadian Centre for Applied Research in Cancer Control (ARCC), BC Cancer Research Centre, 675 West 10th Avenue, Vancouver, BC V5Z 1L3, Canada; 3 School of Population and Public Health, University of British Columbia, 2329 West Mall, Vancouver, BC V6T 1Z4, Canada; 4 Department of Psychiatry, University of Melbourne, Level 1 North Main Block, Royal Melbourne Hospital, Melbourne, VIC 3050, Australia; 5 Public Health Innovation, Deakin Population Health Strategic Research Centre, Deakin University, 221 Burwood Highway, Burwood, VIC 3125, Australia; 6 Centre for Program Evaluation, University of Melbourne, 100 Leicester Street, Parkville, VIC 3052, Australia; 7 Prof Jeff Richardson, Foundation Director, Centre for Health Economics, Monash University, Wellington Road, Clayton, VIC 3800, Australia

**Keywords:** Quality of Life, MAU instrument, HRQoL, Economic evaluation

## Abstract

**Background:**

Multi attribute utility (MAU) instruments are used to include the health related quality of life (HRQoL) in economic evaluations of health programs. Comparative studies suggest different MAU instruments measure related but different constructs. The objective of this paper is to describe the methods employed to achieve content validity in the descriptive system of the Assessment of Quality of Life (AQoL)-6D, MAU instrument.

**Methods:**

The AQoL program introduced the use of psychometric methods in the construction of health related MAU instruments. To develop the AQoL-6D we selected 112 items from previous research, focus groups and expert judgment and administered them to 316 members of the public and 302 hospital patients. The search for content validity across a broad spectrum of health states required both formative and reflective modelling. We employed Exploratory Factor Analysis and Structural Equation Modelling (SEM) to meet these dual requirements.

**Results and Discussion:**

The resulting instrument employs 20 items in a multi-tier descriptive system. Latent dimension variables achieve sensitive descriptions of 6 dimensions which, in turn, combine to form a single latent QoL variable. Diagnostic statistics from the SEM analysis are exceptionally good and confirm the hypothesised structure of the model.

**Conclusions:**

The AQoL-6D descriptive system has good psychometric properties. They imply that the instrument has achieved construct validity and provides a sensitive description of HRQoL. This means that it may be used with confidence for measuring health related quality of life and that it is a suitable basis for modelling utilities for inclusion in the economic evaluation of health programs.

## Background

Health related Multi-Attribute Utility (MAU) instruments seek to measure the quality of life (QoL) of individuals in a way which allows its inclusion in economic evaluation studies and, in particular, Cost Utility Analyses (CUA). While their defining characteristic is the creation of an index of utility (or strength of preference) they may also be used, like non-utility generic instruments such as the SF-36, to measure health related quality of life (HRQoL) or to provide health profiles. Construction of an MAU instrument involves two tasks: creating a descriptive system - a set of questions whose answers describe a person's health state - and devising a scoring formula, which combines and reduces the answers to a single number which seeks to measure utility. The present paper is concerned with the first of these tasks. The methods used for deriving the scoring formula for the AQoL-6D are described in another paper [1].

To date a small number of MAU instruments have dominated the field. These include the EQ-5D, SF-6D, Health Utilities Index (HUI 1, 2, 3), the 15D, the QWB and the AQoL-4D. Descriptions and comparison of these are given elsewhere [2]. Scores from the instruments vary significantly. In the two largest comparative studies to date [3,4] only an average of 53.3 and 46.2 percent of the variation in each instrument's score was explained by variation in other instruments' scores, despite all purporting to measure the same variable- utility - and instrument questionnaires being completed by the same individual at the same point in time. A recent review suggests that the main reason for this is the variable content of items and dimensions [5]. These reflect differences in the researchers' conceptualisation of health, the scope of the concept and the detail of the descriptions. The differences suggest the need for further developmental work into the achievement of content validity. This depends upon the process of instrument construction and psychometric methods have been developed to maximise the likelihood of achieving content validity [6,7]. Despite this, none of the most widely used MAU instruments employed these methods.

The Assessment of Quality of Life (AQoL) program, described in Table 1, sought to increase content validity for health states close to full health and in health dimensions where existing MAU instruments appeared, *prima facie*, to lack sensitivity. A review of the literature suggested that these included health states which are sensitive to the social context ('handicap' i.e. activity/participation) [8]. To achieve this goal, the AQoL construction employed the psychometric methods recommended by instrument construction theory.

**Table 1:** AQoL Instruments

Instrument	Description
AQoL-4D	Originally called 'AQoL': Initially a 5 dimension, 15 item instrument. Dimensions were illness, independent living, social relationships, physical senses, psychological wellbeing. Illness was subsequently deleted from the formula. Utilities are combined with a multi-level model using multiplicative models for dimensions and an overall multiplicative model to combine them [8].

AQoL-8	An 8 item (Brief) instrument which removes one item per dimension from AQoL-4D and imputes their values from remaining items [9].

AQoL-6D	(Originally called AQoL II) A 6 dimensional, 20 item instrument described in this article. Utility weights are constructed as for AQoL-4D but with an econometric adjustment for the final algorithm [1].

AQoL-7D	A 7 dimension 26 item instrument which adds an explicit dimension for vision (VisQoL). Scaling is carried out as for AQoL-6D [10,11].

AQoL-8D	An 8 dimensional 35 item instrument which adds 15 items and 2 dimensions related to mental and social health [12,13].

## Methods

### Overview

The broad steps recommended by psychometric instrument construction theory and adopted by the AQoL-6D are outlined in Table 2. The first step is to determine the overall theory of HRQoL to be embodied in an instrument. This determines the type of items that are subsequently included in the item bank. Secondly, an item bank is constructed (Step 2) and items selected from it which are administered to the target population in a 'construction survey' (Step 3). Final item selection is based upon a statistical analysis of responses - Step 4 - in order to obtain combinations of items which provide a sensitive but parsimonious description of dimensions. AQoL-6D employed a combination of explanatory factor analyses and structural equation modelling (SEM). The latter permits the testing of a pre-specified or imposed theoretical structure to determine whether it satisfies the requirements of a reliable and coherent instrument as indicated by the accepted diagnostic statistics.

**Table 2:** Steps in constructing an MA descriptive system

Step	Activity
1. Theory	Select a Concept of HRQoL (handicap/disability/impairment) Hypothesise dimensions, elements of HRQoL

2. Item Bank	Select items describing dimensions and elements using the literature, focus groups and expert input. Triage items

3. Survey	Carry out a 'construction survey'. Include respondents who have experienced the relevant health states

4. Analysis	Use statistical analyses and judgment for derivation of the final instrument questionnaire

### Theory

Like the earlier AQoL-4D, the AQoL-6D is based upon the hypothesis that (dis)utility depends predominantly upon the effects of a health condition upon a person's capacity to achieve a productive and fulfilling life in their social environment; that is, it conceptualises health primarily in terms of handicap. This is described by the WHO as "a disadvantage for a given individual, resulting from an impairment or disability... (which) limits or prevents the fulfillment of a role that is normal... for that individual" [14] p29]. Thus, for example, the loss of an eye (impairment) may result in disability (an inability to drive) which may result in social handicap (the person may become isolated in their community). Conversely, a blind person who has adapted to their circumstances and, for example, uses a taxi to maintain social contacts, may suffer relatively little loss of utility. More recently the term 'handicap' has been described by the terms 'activity' and 'participation'.

### Item bank

The AQoL-4D item bank included items obtained from a review of published health related instruments, from focus groups and from expert analyses [8]. The AQoL-6D database built upon this, adding items describing positive health states and health states close to full health derived from focus groups, the literature or suggested by the research team. The groups included participants associated with health promotion activities and potential users of the final instrument - clinicians, researchers, and decision-makers. Analysis of transcriptions resulted in new items which were added to the item bank. The research team triaged items to eliminate obviously ambiguous or purely repetitive questions.

### Construction survey

The construction survey provided a set of completed items for statistical analysis to determine the final content and structure of the AQoL-6D. Four main criteria determined item selection: (i) that items should be structurally similar (item stem and response levels) to AQoL-4D items to allow for comparison of results; (ii) that content should be similar to the AQoL-4D as the purpose was an improved instrument covering broadly, the same dimensions of health related QoL; (iii) that items should improve sensitivity; and (iv) that there should be multiple items describing in different ways each of the hypothesised elements shown to be important by the empirical results to this point. Each item included a rating scale to indicate item importance for a person's QoL.

We administered the survey to selected members of the adult population aged 18 and above and to hospital patients in Victoria, Australia. Patient groups were included to increase the likelihood that responses would include the more severe health states described by the items.

The population sample was selected in two stages. In the first, telephone addresses were selected from a computerised directory stratified by postcode, population of postcode and the social economic status of the postcode. A random start sampling interval (RSSI) procedure was used in the case of postcodes with populations that were too small to otherwise warrant inclusion in the sample. In the second stage an introductory letter was followed by a telephone call, a mailed questionnaire and up to two reminder letters or phone calls.

Inpatients were selected from four wards of a major Melbourne teaching hospital. Inpatients and outpatients were initially approached by clinical staff with an explanatory statement and consent form. Interviews were conducted by members of the research team.

The questionnaire was separated into 8 sequences to offset question order effects. Data from the completed questionnaires were double entered and inconsistencies checked and re-entered.

### Initial selection of items

Items were discarded if they had insufficient variance to detect change, if they were expected to discriminate between hospital and population respondents but failed to do so, or if the response level selected constantly conflicted with the importance rating on the accompanying rating scale. Other items were excluded during the subsequent analysis.

### Analysis

Because data from the items was ordinal, the search for instrument structure employed polychoric correlations. Structural equation modelling (SEM) was conducted in two broad stages using the LISREL statistical package. Initially, analysis was conducted dimension by dimension. For example, all items associated with 'social relations' were examined, then items relating to 'independent living' and so forth. Items were excluded which significantly cross-loaded between dimensions. Secondly, the internal structure of each dimension was examined. The objective was to select a small number of items (3-4) which retained a strong substantive coverage of all of the relevant items from the construction survey.

More specifically, the modelling of dimensions was carried out in four steps. The first step was the construction of a model which included all of the items initially included in the construction survey to measure a construct (dimension). Secondly modification indices provided by LISREL were examined to determine additional items. These were added to the model when they improved the fit. Paths were identified by the existence of a correlation between items after taking account of the underlying latent characteristic.

Thirdly, the correlated errors in the resulting model were used to further search for structure among the items, and particularly for "subsets" of variables that could be shown to be measuring the same specific aspects of the dimension (for instance sexuality within the social dimension). Variables were excluded at this stage (e.g. when a single variable achieved a fit which was not significantly inferior to the model which included a multi item structure.

Finally, the remaining items were analyzed (using EQS) to determine Raykov's composite scale reliability, within each dimension. This assisted in further identifying redundant items and, together with the distributional characteristics of responses and the level of missing data, guided the selection of the final set of items. Raykov's composite scale reliability overcomes shortcomings in Cronbach's Alpha which underestimates the coefficient when items are not Tau equivalent, that is to say, when they are differentially related to the underlying construct [15].

The process was iterative and eventually identified items that formed a robust but parsimonious scale within each dimension. These were then combined into an overall model and tested to ensure that each dimension loaded onto a single latent variable, i.e. health related quality of life.

Missing data were dealt with using the Expectation Maximisation (EM) process of the SPSS missing values procedure. This is a form of SEM, which utilises a process similar to estimation by regression, but using latent variables constructed from those items for which responses are available. Variables must be specified for the analysis on a priori grounds such as the substantive context of the items. In effect, the technique allows for errors on the predictor, as well as on the predicted variables. Day et al. [16] provide details of the sampling and analysis.

## Results

### Focus groups and item bank

We conducted four focus groups and combined results with those obtained during the construction of the AQoL-4D. Two groups included health professionals and two employed members of the public. In total, 22 individuals participated in the new groups. All groups identified intrinsic and mediating factors as important for HRQoL, i.e. factors describing the self, and factors facilitating the achievement of HRQoL. We added the former group to the item bank.

The two focus groups which included health professionals sought to identify elements which would be of increasing importance for future HRQoL. Some anticipated the increased importance of less material elements in health, for example spi rituality and sexual fulfillment, while others believed that there would be a movement away from individual level objectives towards the achievement of collective goals. These results led to the addition of new items in the item bank.

Item analysis and triage resulted in the inclusion of 112 items in the questionnaire, a ratio of approximately 5:1 with the targeted instrument size of 15-25 items.

### Instrument questionnaire construction

Three hundred and sixteen (316) members of the general public completed and returned the construction questionnaire, a response rate of 31 percent of the sample initially targeted, 44 percent of the in-scope respondents and 78 percent of those sent a questionnaire. None of the 206 outpatients and 96 inpatients approached by interviewers refused to participate. Table 3 classifies the 618 final respondents by their age, gender, socio-economic characteristics and marital status.

**Table 3:** AQoL-6D Construction survey and questionnaire respondent characteristics

	Male			Female			Total
Community	132			184			316

Hospital	156			148			304

Total	288			332			620

**Sample Group**		**Community**			**Hospital**		
		
		**Male**	**Female**	**Total**	**Male**	**Female**	**Total**

							
Age	18-24 years	3	9	12	4	6	10
	
	25-34	12	33	45	12	14	26
	
	35-44	18	39	57	16	30	46
	
	45-54	30	39	69	12	24	36
	
	55-64	33	34	67	52	34	86
	
	64+	36	30	66	60	40	100
	
	Total	132	184	316	156	148	304

Education	primary	19	11	30	32	22	54
	
	high school	36	72	108	82	84	166
	
	trade	28	16	44	12	14	26
	
	university	43	73	116	22	24	46
	
	other	6	12	18	8	4	12

Occupation	full time	63	38	101	40	12	52
	
	part time	13	58	72	8	24	32
	
	unemployed	7	2	9	10	6	16
	
	home	2	33	34	2	36	38
	
	retired	43	48	91	72	50	122
	
	student	2	3	5	2	4	6
	
	other	1	3	4	20	18	38
	
	Total	131	185	316	154	150	304

Marital status	married/de facto	99	132	231	108	62	170
	
	single/never married	19.5	18	37	26	30	56
	
	single/widowed	4.5	16	21	4	16	20
	
	single/divorced	9	18	27	18	40	58
	
	Total	132	184	316	156	148	304

Income	Under $20,000	42	40	82	62	86	148
	
	20,001-30,000	22	22	45	32	16	48
	
	30,001-40,000	15	19	34	14	8	22
	
	40,001-50,000	10	22	33	2	6	8
	
	50,001-60,000	16	24	40	18	0	18
	
	60,001-80,000	15	10	25	2	10	12
	
	above 80,000	11	31	42	6	4	10
	
	Total	131	168	301	136	130	266
	
	Missing	7	8		20	18	

Table 4 summarises the final instrument and the questionnaire is reproduced in Additional file 1: Appendix 1. Questionnaires included few missing items (questions not answered), except for the three questions about intimate relationships. These items were subjected to Expectation Maximisation Estimation using SPSS Missing Values procedure [17].

**Table 4:** Summary of the AQoL-6D content

Items	Item summary	Number of response categories	Response range
	**Dimension 1 Independent Living**		

1	help with household tasks	5	no help, efficient ↔ can do none

2	mobility outside house	6	easy, enjoyable ↔ need help

3	walking	6	running easy ↔ bedridden

4	self care	5	very easy ↔ need help

	**Dimension 2 Relationships**		

5	intimate relationships	5	very happy ↔ very unhappy

6	health and family role	4	no affect ↔ incapacity

7	health and community	4	no affect ↔ incapacity

	**Dimension 3 Mental Health**		

8	despair	5	never ↔ all the time

9	worry	5	never ↔ all the time

10	sadness	5	never ↔ all the time

11	calm, agitation	5	always calm ↔ always agitated

	**Dimension 4 Coping**		

12	energy	5	always energetic ↔ always tired

13	control of life	5	always ↔ never

14	coping with problems	5	completely ↔ not at all

	**Dimension 5 Pain**		

15	frequency	4	rarely ↔ most of the time

16	discomfort	4	none ↔ unbearable

17	interference with activities	5	never ↔ always

	**Dimension 6 Senses**		

18	vision	6	excellent ↔ blind

19	hearing	6	excellent ↔ deaf

20	communication	4	no trouble ↔ cannot communicate

**Figure 1:**
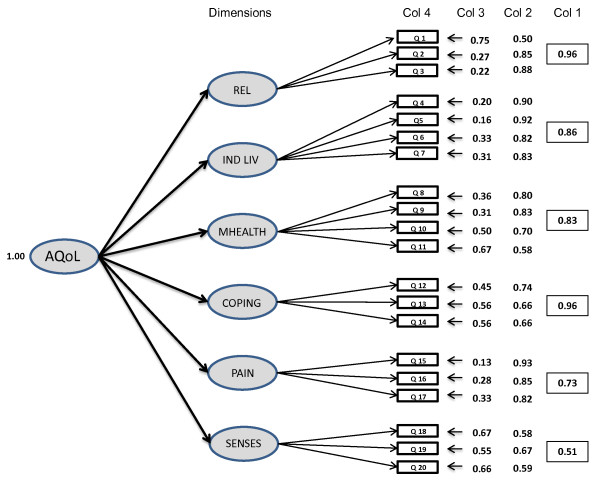
**Structure of the AQoL-6D descriptive system**.

Figure 1 reports the instrument structure and results of the SEM analysis. The instrument consists of 6 dimensions and 20 items. Each of these has between 4 and 6 response levels. Commencing on the right side of Figure 1, the first column of numbers (in boxes) are the gamma coefficients between the dimensions and AQoL latent variables. These are equivalent to standardised correlation coefficients. With the exception of sensory perception where the gamma coefficient is 0.51, all of the coefficients are 0.73 or greater. Lambda weights between the observed item responses and the dimension latent variables - the middle column of Figure 1 - are also equivalent to correlation coefficients. None is below 0.50. Error terms on the individual items in the final, left hand column, are generally low for an analysis of individual level data.

The six dimensions all load on the one HRQoL factor, while the results of the SEM indicate that they are measuring different aspects of HRQoL. This suggests that the items form a uni-dimensional, parsimonious scale. Where data deviate substantially from normality Yu [18] recommends a Comparative Fit Index (CFI) above 0.95 and RMSEA statistic around 0.5, with a maximum of 0.6 for acceptable Type I and Type II errors. The overall Comparative Fit Index (CFI) for the AQoL-6D of 0.97 is higher than the criterion of 0.95 recommended by Yu. The RMSEA of 0.054 is well below 0.08, the generally accepted maximum criterion for a satisfactory fit and within Yu's range for acceptable error. In sum, using the accepted statistical criteria, [19] the results summarised in Figure 1 indicate an exceptionally good result and represent strong evidence of the validity of the model as a representation of the data from the construction sample.

## Discussion

This paper described the methods used to achieve content validity in the AQoL-6D and, particularly, to increase instrument sensitivity as compared with its predecessor the AQoL-4D. To achieve this we employed psychometric methods. We constructed an item bank using the results of earlier studies supplemented by the results from additional focus groups. After initial triage, patients and members of the public were asked to complete the items - the 'construction survey'. Analysis of the 618 sets of completed items employed polychoric correlations and structural equation modeling to optimise the number of items in each of the postulated dimensions while simultaneously determining the configuration of dimensions which best represented the postulated concept of HRQoL.

This latter task encounters a particular challenge which arises from the distinction between formative and reflective modelling. In the latter, causation runs from the latent (unobserved) variable to the items. (For example, it might be hypothesised that a latent variable for 'intelligence' is causally related to observed items measuring arithmetic and linguistic achievements, as intelligence *causes *high achievement.) With formative models causation is reversed [20]. For example, socio economic status (SES) does not cause education or income. Rather, education and income, *inter alia*, define SES.

In the AQoL-6D there are elements of both types of model. This was handled analytically through the use of a multi level descriptive system. The relationship between dimensions and items is largely reflective. 'Coping' results in energy, control and problem solving. However the relationship between dimensions and the overall HRQoL construct is largely formative. HRQoL summarises the shared variance between the dimensions which define HRQoL and it would not, for example, be conceptually correct to omit dimensions as a result of a poor loading obtained from exploratory factor analysis. SEM permits both forms of modelling to occur simultaneously: the best set of indicator items for achieving content validity may be obtained subject to constraints placed upon the overall structure of the model.

For this reason, at all stages of the analysis, the structure of the model was reviewed from a substantive as well as a statistical viewpoint. Decisions between various models were informed by statistical considerations but also by a consideration of what is generally regarded as HRQoL. For example, while loadings between items and dimensions (lambdas) are generally high, the loading on question 18 is only 0.58. This suggests that vision is less closely related to HRQoL than most other items in the model. This is readily interpretable: people can suffer poor vision without it being related to other deficiencies in HRQoL. However, to exclude vision from a generic instrument would violate the usual understandings of HRQoL, and, consequently, it is included in the model. Despite the low loading it does not compromise the overall fit of the model.

Successful application of these methods resulted in a relatively large instrument. Its 20 items define 5.4 × 10^13 ^health states. This does not guarantee content validity, but the methods employed increase the likelihood that this has been achieved. The *prima facie *evidence for this is presented in Table 5 which compares the number of items in the most widely used MAU instruments by broad area of health. Table 5 also indicates that there is a highly imperfect overlap between the item content of different instruments and that, even with its larger numbers, the AQoL-6D does not include some items which are included in some other instruments. In particular it does not include breathing, sleeping, eating and elimination as does the 15D, or dexterity, cognition and memory as does the HUI 3. Conversely, all of the other instruments omit items included in the AQoL-6D.

**Table 5:** Comparison of the dimensions and content of 6 MAU instruments

		Number of items in MAU instruments (*)					
	**Dimension**	**15D**^**(1)**^	**EQ-5D**	**HUI 3**	**SF-6D**	**AQoL-4D**	**AQoL-6D**

**Physical**	Physical ability/vitality/coping/	*			*		***
	
	Bodily Function/Self Care	***	*			*	**
	
	Dexterity			*			
	
	Pain/Discomfort	*	*	*	*	*	**
	
	Senses	**		**		**	**
	
	Usual activities/Work function	*	*	*	*		
	
	Mobility/walking	*	*	*		*	**
	
	Communication	*		*		*	*

**Psycho-social**	Sleeping	*					
	
	Psychological: Depression/Anxiety/Anger	***	*	*	*	*	*
	
	Satisfaction/Happiness						****
	
	Cognition/Memory Ability			*			
	
	Social Function/Relationships				*	**	*
	
	(Family) Role				*	*	*
	
	Intimacy/Sexual Relationships	*					*

		15 items	5 items	8 items	12 items	12 items	20 items

(Each asterisk represents a single item)

(1) 15D also includes breathing, sleeping, eating and elimination

The differences are the result of two decisions made during construction of each instrument. The first is the conceptual basis of the instrument. As described earlier, AQoL instruments sought to model 'handicap' (activity/participation). In contrast, the 15D and HUI 3 focused upon body function and structures (a 'within the skin' framework). Their items, including those noted above (breathing, etc.) are important but in a handicapped based instrument these items are, in principle, reflected in the consequences of body impairment for handicap.

The second decision is the extent of the trade-off between sensitivity and parsimony. All instruments necessarily limit the number of items and some elements of a health state are necessarily under-represented. In principle, the choice of items should be made on the basis of the psychometric evidence relating to the goodness of fit of the alternative combinations of items. This has been the subject matter of the present article. The success of the modelling, as compared with the alternative models must be determined by comparative studies.

The existence of multiple instruments, including multiple AQoL instruments, raises three questions. The first is why more than one 'generic' instrument is necessary. From the evidence of comparative studies cited in the introduction MAU instruments (and not just AQoL instruments) produce different scores. One of two conclusions must therefore be drawn: either different instruments have a differing capacity for detecting utility in different contexts or, all of the instruments with one exception, create scores which do not correspond with utility. To date this latter possibility remains unproven and the evidence supports the former conclusion. From its construction and the evidence in Table 5 the AQoL-6D would be expected to achieve greater sensitivity than other instruments in some, but not all of the dimensions it includes. The variability of the combinations of items in Table 5 suggests that different instruments may achieve greater sensitivity in different contexts. For example, the HUI 3 might achieve greater sensitivity for health states dominated by problems with dexterity or cognition.

The strength of the AQoL instruments relative to other MAU instruments to date is that they have been derived using psychometric procedures, which increases the evidence for reliability and validity in the dimensions of health covered by the instruments. This does not, however, guarantee the universal superiority of the instruments. Context specific strengths and weaknesses of instruments require independent empirical research and a recent review of this literature indicates that this comparative research has not been carried out satisfactorily for the other major instruments [5]. The evidence of construct validity presented here is therefore of particular importance.

The second question is how scores from different instruments might be compared. One approach to the question is to side-step the problem by only using a single instrument. However the result would be analogous to the use of a single diagnostic test, for example, blood pressure, to compare the severity of all diseases. Comparisons would not be valid without the demonstration of a universal 'best' generic instrument. Part of the final answer is likely to include the creation of statistical transformations between instrument scores as has been done between the AQoL instruments [5]. However, aligning the measurement units is necessary but not sufficient for comparability and transformations per se cannot inject sensitivity into insensitive instruments.

This raises the third question of which MAU instrument should be used. As suggested above there is, at present, no general answer implying that researchers must judge which instrument is most likely to detect program specific changes in the quality of life. Economic evaluation of health programs, in particular, requires the inclusion of all utility-relevant information in the description of health states and the omission of this will compromise the validity of the measurement. The AQoL-6D was a response to evidence that the sensitivity of existing instruments is imperfect. The extent to which the resulting instrument meets this need is a matter for further research.

## Conclusions

We created the AQoL-6D as the descriptive system for an MAU instrument. While the utility algorithm is available on the AQoL website [21] the instrument may be used with or without utility weights. Utility scores are necessary for an economic evaluation which employs Quality Adjusted Life Years (QALYs). For other purposes unweighted results might suffice or be superior, as utility algorithms create highly skewed distributions.

The comparative strengths of existing MAU instruments have not been satisfactorily documented. This implies that individual researchers must choose between them. The primary criterion is that an instrument can, *prima facie*, detect the changes in health states which the researcher expects to observe. The breadth of the AQoL-6D implies that it should rate highly on this criterion. The evidence presented in this paper should also increase confidence in the validity and reliability of the instrument.

Despite this conclusion, the AQoL-6D is a relatively new instrument and further research is needed to establish its validity in specific contexts. In particular, it would be desirable to use the instrument in tandem with a disease specific instrument and, preferably, with another generic QoL instrument. This maximises confidence in particular results and assists with the process of validating the AQoL-6D in that context.

## Competing interests

The authors declare that they have no competing interests.

## Authors' contributions

JR, Original conceptual design; primary responsibility writing the paper. SP, Assistance with interpretation of data. GH, Conceptual input; interpretation of data. AI, Project management; data collection. GE, Interpretation of data; assistance with analysis. ND, Conceptual input; statistical analysis. All authors read and approved the final manuscript.

## Supplementary Material

Additional file 1 **Appendix 1 AQoL-6D Questionnaire**. Click here for file
