# Supplementary material for: Construction of the descriptive system for the assessment of quality of life AQoL-6D utility instrument
Source: Health Qual Life Outcomes. 2012 Apr 17;10:38. doi: 10.1186/1477-7525-10-38 (PMC3349491; doi:10.1186/1477-7525-10-38)
Supplement: Additional file 1 — Appendix 1 AQoL-6D Questionnaire. [file 1477-7525-10-38-S1.DOCX]

Additional file 1: Appendix 1 AQoL-6D Questionnaire

Dimension 1: Independent Living

**Q1** How much help do I need with household tasks (e.g. preparing food, cleaning the house or gardening):

| □ | I can do all these tasks very quickly and efficiently without any help |
| --- | --- |
| □ | I can do these tasks relatively easily without help |
| □ | I can do these tasks only very slowly without help |
| □ | I cannot do most of these tasks unless I have help |
| □ | I can do none of these tasks by myself. |

**Q2** Thinking about how easy or difficult it is for me to get around by myself outside my house (e.g. shopping, visiting):

| □ | getting around is enjoyable and easy |
| --- | --- |
| □ | I have no difficulty getting around outside my house |
| □ | a little difficulty |
| □ | moderate difficulty |
| □ | a lot of difficulty |
| □ | I cannot get around unless somebody is there to help me. |

**Q3** Thinking about how well I can walk:

| □ | I find walking or running very easy |
| --- | --- |
| □ | I have no real difficulty with walking or running |
| □ | I find walking or running slightly difficult. I cannot run to catch a tram or train, I find walking uphill difficult |
| □ | walking is difficult for me. I walk short distances only, I have difficulty walking up stairs |
| □ | I have great difficulty walking. I cannot walk without a walking stick or frame, or someone to help me |
| □ | I am bedridden. |

**Q4** Thinking about washing myself, toileting, dressing, eating or looking after my appearance:

| □ | these tasks are very easy for me |
| --- | --- |
| □ | I have no real difficulty in carrying out these tasks |
| □ | I find some of these tasks difficult, but I manage to do them on my own |
| □ | many of these tasks are difficult, and I need help to do them |
| □ | I cannot do these tasks by myself at all. |

Dimension 2: Relationships

**Q5** My close and intimate relationships (including any sexual relationships) make me:

| □ | very happy |
| --- | --- |
| □ | generally happy |
| □ | neither happy nor unhappy |
| □ | generally unhappy |
| □ | very unhappy |

**Q6** Thinking about my health and my relationship with my family:

| □ | my role in the family is unaffected by my health |
| --- | --- |
| □ | there are some parts of my family role I cannot carry out |
| □ | there are many parts of my family role I cannot carry out |
| □ | I cannot carry out any part of my family role. |

**Q7** Thinking about my health and my role in my community (that is to say neighbourhood, sporting, work, church or cultural groups):

| □ | my role in the community is unaffected by my health |
| --- | --- |
| □ | there are some parts of my community role I cannot carry out |
| □ | there are many parts of my community role I cannot carry out |
| □ | I cannot carry out any part of my community role. |

Dimension 3: Mental Health

**Q8** How often did I feel in despair over the last seven days?

| □ | never |
| --- | --- |
| □ | occasionally |
| □ | sometimes |
| □ | often |
| □ | all the time. |

**Q9** And still thinking about the last seven days: how often did I feel worried:

| □ | never |
| --- | --- |
| □ | occasionally |
| □ | sometimes |
| □ | often |
| □ | all the time. |

**Q10** How often do I feel sad?

| □ | never |
| --- | --- |
| □ | rarely |
| □ | some of the time |
| □ | usually |
| □ | nearly all the time. |

**Q11** When I think about whether I am calm and tranquil or agitated:

| □ | always calm and tranquil |
| --- | --- |
| □ | usually calm and tranquil |
| □ | sometimes calm and tranquil, sometimes agitated |
| □ | usually agitated |
| □ | always agitated. |

Dimension 4: Coping

**Q12** Thinking about how much energy I have to do the things I want to do, I am:

| □ | always full of energy |
| --- | --- |
| □ | usually full of energy |
| □ | occasionally energetic |
| □ | usually tired and lacking energy |
| □ | always tired and lacking energy. |

**Q13** How often do I feel in control of my life?

| □ | always |
| --- | --- |
| □ | mostly |
| □ | sometimes |
| □ | only occasionally |
| □ | never. |

**Q14** How much do I feel I can cope with life’s problems?

| □ | completely |
| --- | --- |
| □ | mostly |
| □ | partly |
| □ | very little |
| □ | not at all. |

Dimension 5: Pain

**Q15** Thinking about how often I experience serious pain. I experience it:

| □ | very rarely |
| --- | --- |
| □ | less than once a week |
| □ | three to four times a week |
| □ | most of the time. |

**Q16** How much pain or discomfort do I experience:

| □ | none at all |
| --- | --- |
| □ | I have moderate pain |
| □ | I suffer from severe pain |
| □ | I suffer unbearable pain. |

**Q17** How often does pain interfere with my usual activities?

| □ | never |
| --- | --- |
| □ | rarely |
| □ | sometimes |
| □ | often |
| □ | always |

Dimension 6: Senses

**Q18** Thinking about my vision (using my glasses or contact lenses if needed):

| □ | I have excellent sight |
| --- | --- |
| □ | I see normally |
| □ | I have some difficulty focusing on things, or I do not see them sharply. E.g. small print, a newspaper or seeing objects in the distance. |
| □ | I have a lot of difficulty seeing things. My vision is blurred. I can see just enough to get by with. |
| □ | I only see general shapes. I need a guide to move around |
| □ | I am completely blind. |

**Q19** Thinking about my hearing (using my hearing aid if needed):

| □ | I have excellent hearing |
| --- | --- |
| □ | I hear normally |
| □ | I have some difficulty hearing or I do not hear clearly. I have trouble hearing softly-spoken people or when there is background noise. |
| □ | I have difficulty hearing things clearly. Often I do not understand what is said. I usually do not take part in conversations because I cannot hear what is said. |
| □ | I hear very little indeed. I cannot fully understand loud voices speaking directly to me. |
| □ | I am completely deaf. |

**Q20** When I communicate with others, e.g. by talking, listening, writing or signing:

| □ | I have no trouble speaking to them or understanding what they are saying |
| --- | --- |
| □ | I have some difficulty being understood by people who do not know me. I have no trouble understanding what others are saying to me. |
| □ | I am understood only by people who know me well. I have great trouble understanding what others are saying to me. |
| □ | I cannot adequately communicate with others. |
